# Articulating conjunctural analysis

**DOI:** 10.1177/20438206241242471

**Published:** 2024-04-07

**Authors:** Jamie Peck

**Affiliations:** University of British Columbia, Canada

**Keywords:** Collaboration, conjunctural analysis, methodology

## Abstract

In dialogue with responses to my article on the emergent practice of conjunctural methodologies, I pick up the question of collaboration and the shared challenges of developing, in a deliberative and reflexive manner, this demanding approach to problem specification, research design, and contextual theorizing. Although explicit engagement with conjunctural methodologies is a relatively recent phenomenon, its connections and resonances with geographical research practice run deeper. This means that there is much for geographers to give as well as to gain from the interdisciplinary conversation around conjunctural analysis.

First of all, I would like to thank my interlocutors here – Han Cheng, Allan Cochrane, Ruben Gonzalez-Vicente, Colin Lorne, Shaun Teo, Matthew Thompson, and Henry Yeung – for their generous and constructive engagement. I am not sure that there is a great deal that we disagree on, if anything, even if some of this is down to a shared sense of the challenges (if also the promise) of conjunctural analysis in and for critical human geography. The commentaries each raise questions relating to operational practicality and political purpose that are undeniably real and pressing, while also pointing to the need for, and returns to, what are effectively collaborative responses. The exchange also broadly confirms the lingering sense that, even if the language of conjunctural analysis has been circulating in critical human geography for several years now, the approach remains aspirational and in some respects elusive. On the face of it, there is a compelling degree of ‘fit’, or congruence, between what many critical human geographers do, and the precepts of conjunctural analysis (grounded and situated investigations, executed on moving terrains; contextual and reflexive theorizing, married to a disruptive ethos and commitments to politics otherwise; distrust of economism, functionalism, and parsimonious, reductive or monocausal explanation, etc.). On the other hand, there is a sense that the adoption of conjunctural methodologies in critical human geography might still be a stretch; that in some respects it might even be out of reach, too onerous, or just too much.

It is quite possible, however, to invoke another conjuncturalist principle, that more than one thing can be going on at the same time. There are strong (and arguably defining) connections between conjunctural analysis and the work of political-economic geographers, such as Doreen Massey, John Pickles, Adrian Smith, Gillian Hart, Vinay Gidwani, and Marion Werner. There are rich traditions, too, of contextual theorizing, in a variety of registers (see Lewis, 2024; Yeung, 2024). Furthermore, the discipline possesses a methodological atmosphere that favors (indeed tends to reward) innovation, openness, and creativity, although this has not necessarily translated into transparency and reflexivity around questions of epistemological strategy, modes of theorizing, research design and practice, and so forth (see Barnes et al., 2007; Barnes and Christophers, 2018). In this latter respect, there is much that remains unsaid, or barely articulated. Perhaps ironically, something similar might be said of conjunctural analysis, which has been demonstrated more in execution than through explicit methodological elucidation. This remains an approach, critical disposition, or ethos that is not reducible to an entirely codifiable method or a portable toolkit of replicable routines (Hart, 2023; Sheppard et al., 2024), despite practically overflowing with methodological implications and perhaps obligations – such that the implied ‘search for method’ has been a long, continuing, and largely retrospective one (Clarke, 2020, 2023; Grossberg, 2019). In this somewhat daunting context, the more limited goal of my *Dialogues* paper was to pin down aspects of the method, qua method, from a perspective and position in geographical political economy, moving from matters to principle to more grounded issues of practice, with reference to an ongoing research collaboration centered on China's Greater Bay Area (see Anguelov et al., 2024; Ebner and Peck, 2022; Meulbroek et al., 2023; Peck, 2021; Peck et al., 2023, 2024). This is not to imply, just to be clear, that a full-service methodological ‘solution’ is at hand in this ongoing project. However what can be said is that the attendant questions of research design, specification, and practice are being engaged explicitly and frequently.

This context matters, because the methodological challenges, responses, and strategies outlined in my *Dialogues* paper (Peck, 2024) are somewhat specific to the evolving research program in geographical political economy, and in some ways particular to the (Chinese) global-regional context too. It perhaps goes without saying that these are necessary caveats. Conjunctural analysis is not an all-purpose replacement, or comprehensive substitute, for all other methods. As an approach to ‘analyzing situations’, it is not universally suitable for every situation. This said, there are also methodological implications that are quite particular, especially concerning matters of research design, case specification, explication, and exegesis, from the question of where to begin to how then to proceed. Furthermore, conjunctural analysis does not provide answers to each and every explanatory and interpretive problem, even as it does offer a particular way of confronting challenges of problem specification, theorization, and explanation. Finally, conjunctural analysis is not a hermetically sealed or complete methodology, neatly sequestered from all other approaches, but exhibits affinities with ‘intensive’ or qualitative-interpretative methods, and with some varieties of ethnography, with critical discourse analysis, and with the extended case method. As such, it exists less as a bloodless set of rules and more as an evolving form of craft practice; its work is always in progress.

There are reasons, consequently, to name conjunctural analysis, and to invite ongoing dialogue around its specification and implications, within and between contexts. For my own part, while I have been chipping away at the question of how to operationalize conjunctural analysis for about a decade now (Peck, 2015, 2017), it has been an imminent presence for much longer. Polanyian methodologies, for example, are predicated on the recognition of ‘local’ socioeconomic heterogeneity, coupled with an invitation to interrogate part-whole connections, relations, and interactions across various ‘comparative’ registers (Peck, 2013b). The geography of actually existing markets, to mention another long-time concern, is likewise more than a product of merely contingent variation around some tendentially universal logic, but instead resembles a mosaic of institutionally embedded configurations, unevenly crosscut by processes of regulation and marketization (Berndt et al., 2020; Peck, 1996, 2020). The ontological conditions of the existence of neoliberalization, as a simultaneously reactive and proactive modality of market rule, are themselves conjunctural, since neoliberalism can only exist in contradictory hybrids, in a volatile combination with its others (Peck, 2013a; Peck and Theodore, 2012; Peck and Whiteside, 2018). And the landscape of variegated capitalism is shaped by the unstable coexistence of a plurality of recombinant forms, the uneven and interdependent development of which really does play a constitutive role (Dixon et al., 2023; Peck, 2023). The latter, in fact, provided a starting premise for our project on the capitalist border zones of South China, which is just about the last place to go if the goal is to tidy up ideal-typical models of ‘variety’, but where it is also manifestly insufficient to engage unruly processes of variegation willy-nilly, in some open-ended fishing expedition, sans some kind of theoretically informed ‘map’.

For these and other reasons, then, there is a rationale for the deliberative development of conjunctural methodologies that problematize situated, multiple causality and which approach ‘casing’ in conjunction with the continuing work of theorization. Here, geographers have much to gain from engagements with the rich traditions of conjunctural analysis from across the critical social sciences, but there is also much to give. With some notable exceptions (e.g. Li, 2014), ‘conjunctural analysis … has tended to take a national territorial framing for granted’ (Clarke, 2023: 15), underscoring the need for ‘spatialized’ approaches. These would attend not only to historicization, extending explanatory frameworks beyond the temporally immediate, back through time; they would also reach beyond the spatially proximate, extending explanatory horizons ‘outwards in space (identifying how local events are shaped by distant processes), and upward and downward in terms of geographical scale (whereby events at a particular scale may be shaped by both higher and lower scale processes)’ (Leitner and Sheppard, 2020: 495; Sheppard et al., 2024). It cannot be sufficient, as John Clarke (2023: 15, 29–30) points out, to substitute what has been a tacit methodological nationalism with an alternative methodological globalism, reducing localized sites to ‘scalar diminutives’, or subordinate parts of an overbearing whole. At the same time, there are reasons to be wary of those methodological localisms, for which we geographers tend to have a soft spot, that bracket out the interlocal and uneven spatial development, while ‘capping’ the consideration of (trans)scalar and hierarchical relations. Suggestive here are the approaches to conjunctural regionalism, Chinese style, proposed by Cheng and Gonzalez-Vicente (2024), where these actively engage interregional and interscalar relations, following connections and spiraling out in different ways, including through diasporic networks, across to peer regions, down to competing local interests, or out to the power hierarchies of the party state. On the other hand, if there are risks in formulations like ‘urban China’, as Teo (2024) cautions, it is that they invite sequestered modes of explanation within pregiven parameters, rather than pushing to challenge, exceed, and rework these limits.

To think across cases and contexts, sites and situations, is to theorize conjuncturally, to stress-test competing explanations and to probe alternative sources of conditioning and causation. Practically speaking, this will inevitably exceed the work of individual projects and for that matter individual researchers, being instead the shared and distributed work of research programs, collaborations, debates, and disputes. As Lorne et al. (2024) properly point out, the work conjunctural analysis is necessarily a collective endeavor. (This is another reason, of course, to specify and stress test theory claims, since this is one way to loop our projects into wider conversations, and to interrogate concepts across as well as within contexts. Reflexive theorizing, in this sense, constitutes a grammar of collaboration, or might be thought of as a technology of collaboration.) The proof of these efforts – the collaborative development of conjunctural approaches – will be in the pudding, needless to say, although there is also much to gain from experimentation with different recipes, enriched by methodological reflexivity. There is room for lots of cooks in this particular kitchen.
